# Dammarane triterpenes targeting α-synuclein: biological activity and evaluation of binding sites by molecular docking

**DOI:** 10.1080/14756366.2020.1851216

**Published:** 2020-12-14

**Authors:** Alberto Cornejo, Julio Caballero, Mario Simirgiotis, Vanessa Torres, Luisa Sánchez, Nicolás Díaz, Marcela Guimaraes, Marcos Hernández, Carlos Areche, Sergio Alfaro, Leonardo Caballero, Francisco Melo

**Affiliations:** a Escuela de Tecnología Médica, Facultad de Medicina, Universidad Andres Bello, Laboratorio Catem V, Santiago, Chile; b Departamento de Bioinformática, Facultad de Ingeniería, Centro de Bioinformática, Simulación y Modelado (CBSM), Universidad de Talca, Talca, Chile; c Facultad de Ciencias, Instituto de Farmacia, Universidad Austral de Chile, Valdivia, Chile; d Department of Science and Technology, Federal University of São Paulo, São José dos Campos, Brazil; e Departamento de Química, Facultad de Ciencias, Universidad de Chile, Santiago, Chile; f Doctorado en Ciencias, mención Modelado de Sistemas Químicos y Biológicos, Centro de Bioinformática, Simulación y Modelado (CBSM), Facultad de Ingeniería, Universidad de Talca, Talca, Chile; g Departamento de Física and Soft Matter Research Center, SMAT-C, Universidad de Santiago, Santiago, Chile

**Keywords:** Parkinson’s disease, oligomers, drug target, natural compounds modifiers

## Abstract

Parkinson's disease (PD) is a neurodegenerative disorder that affects adult people whose treatment is palliative. Thus, we decided to test three dammarane triterpenes **1**, **1a**, **1b**, and we determined that **1** and **1a** inhibit β-aggregation through thioflavine T rather than **1b**. Since compound **1** was most active, we determined the interaction between α-synuclein and **1** at 50 µM (Kd) through microscale thermophoresis. Also, we observed differences in height and diameter of aggregates, and α-synuclein remains unfolded in the presence of **1**. Also, aggregates treated with **1** do not provoke neurites' retraction in N2a cells previously induced by retinoic acid. Finally, we studied the potential sites of interaction between **1** with α-synuclein fibrils using molecular modelling. Docking experiments suggest that **1** preferably interact with the site 2 of α-synuclein through hydrogen bonds with residues Y39 and T44.

## Graphical Abstract

**Figure:**
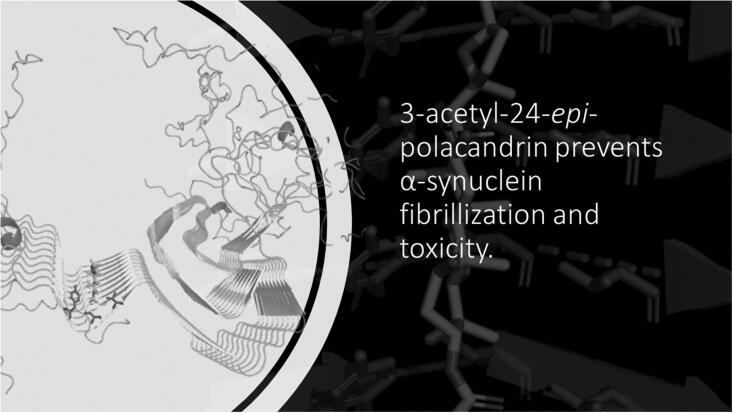

## Introduction

The protein α-synuclein is a 14 kDa protein that belongs to the family of synucleins. It has been associated with neurodegenerative disorders, and it is found in high concentrations in presynaptic terminals, associated with the reserve pool of synaptic vesicles^1^^,^^2^. This protein has been associated with synaptic vesicle recharge and trafficking from the reserve pool to its release, interacting with proteins that control vesicle exocytosis, such as the family of small RAB GTPases, phospholipase D2, and promoting the SNARE complex, which is directly involved in the release of neurotransmitters, including dopamine^3^. Its structure is divided into three regions; the N-terminal region, characterised by having 11 repetition domains of seven amino acids giving as a secondary structure alpha amphipathic helix^4^.

Between the amino acids, 61–95 resides the NAC region, known as the non-amyloid-β component, highly hydrophobic, is indispensable for aggregation. The elimination of large segments of this region dramatically decreases fibrillogenesis. Finally, the C-terminal region is structured by negatively charged amino acids, resulting in a polar region^1^. This protein is encoded on chromosome 4 q21 (SNCA gene)^5^, whose point mutations are located predominantly in the N-terminal region as A30P, E46K, A53T, and some recently described G51D and H50Q, associated with different PD varieties^6^.

Moreover, α-synuclein is prone to form aggregates and subsequently leads to the formation of amyloid deposits. In Parkinson's disease (PD), α-synuclein aggregates can be found in dopaminergic neurons as self-misfolding protein inclusions known as Lewy bodies^7^. More importantly, this folding can form oligomers, which have a toxic effect on the neuronal membrane, altering permeability, causing an influx of calcium, depolarising the membrane. Also, oligomers can cause oxidative damage leading to cell death^8^. This neurodegenerative disorder's hallmark is characterised by the accumulation of intraneuronal inclusions composed of α-synuclein rich in β-sheet. These inclusions' neurotoxicity could influence other proteins to lose their function, having a role structure such as prefibrillar species, which might be toxic to neurons^1^. In PD, the primary treatment is levodopa (l-DOPA). However, several studies have found side effects in patients who use this medication, including nausea, vomiting, and significant movement disorders. These symptoms can increase in frequency if l-DOPA loses its effect. Also, catechol-O-methyl transferase (COMT) inhibitors are a ubiquitous enzyme that catalyses the O-methylation catechol-containing substrates by transferring, in the presence of magnesium, a methyl group from S-adenosylmethionine. The methylation is essential to discard biologically active or toxic catechols and some other hydroxylated metabolites^9^. Moreover, inhibitors such as opicapone have a typical structure: (3,4-dihydroxy-5-nitrophenyl), suggesting that this structure can increase systemic and central l-DOPA bioavailability^10^.

Along with the mechanism of action, substantial evidence indicates that it causes low secondary effects for Parkinson's patients, contrasting with other COMT inhibitors, more commonly used like tolcapone^11^. Moreover, several approaches have risen to focus on the clearance of toxic deposits, promoting proteasomal activity, and targeting the inhibition of α-synuclein aggregates^12^. Interestingly, the polyphenol (–)-epigallocatechin gallate redirects amyloid fibril formation of α-synuclein into a non-pathogenic pathway^13^.

 In the present work, we demonstrated that dammarane triterpene **1** inhibits aggregates progression of α-synuclein (50 µM Kd) through hydrogen bonds from hydroxyl and carbonyl groups of compound **1**. Moreover, α-synuclein, after 48 h of aggregation in the presence of **1** it remains unfolded, demonstrated through Raman spectroscopy experiments. Then, we demonstrated that oligomers in the presence of **1** did not exert neurite retraction in N2a cells instead of aggregates untreated that exert a deleterious effect on cells. Ultimately, we show how **1** interacts with α-synuclein fibrils that interact with different sites, evidentiating that compounds such as dammarane triterpenes may serve as a scaffold for exploring new drug design for PD.

## Materials and methods

### Chemistry

Silica gel (Kieselgel 60, Merck, Darmstadt, Germany, 0.063–0.200 mm) and Sephadex LH-20 were used in column chromatography (CC). Solvents used in extraction were previously distilled and dried according to standard procedures. The extraction and purification of **1**, **1a**, and **1b** were carried out mainly as described^14^. The purity of all compounds was estimated by using an HPLC-PDA (≥98%) (Agilent LC-1200, Santa Clara, CA). α-recombinant protein was purified by HPLC and, ThT assays were performed in a Biotek H1. The dissociation constant (Kd) was determined through microscale thermophoresis (MTS). The atomic force images were obtained using a Nanoscope III equipment in tapping mode. The Raman spectra and maps were obtained through a Confocal Raman Microscopy Alpha 300 (WITec GmbH, Ulm, Germany).

*Extraction and isolation.* The air-dried aerial parts of *Ibicella lutea* from the Province of Mendoza (2.5 kg) were extracted with CHCl_3_ to obtain after evaporation of the solvent, 105 g of extract^14^. The CHCl_3_ extract (50 g) was fractionated by vacuum-liquid chromatography (VLC) over silica gel 60 H (Merck, Darmstadt, Germany). The fraction eluted with 50% EtOAc (5 g) was then chromatographed on HPLC reversed-phase silica gel with MeOH-H_2_O (7:3, v:v), and recrystallisation from CH_3_CN, to afford compound **1** (1β-acetoxi-20(*S*), 24(*R*)-epoxi-3α, 12β, 25-trihidroxidammarane, 230 mg).

*Hydrolysis of compound* **1**. Compound **1** (10 mg) was added to a solution of 10% KOH in MeOH (5 ml) under argon, and the mixture was stirred at room temperature for 8 h. Then, 2 ml of the solvent was evaporated, and the remaining solution was poured into a mixture of 5 g of ice and 10 ml of 1 M HCl. The mixture was extracted with EtOAc (2 × 10 ml). The combined extracts were washed with brine (10 ml), dried over Na_2_SO_4_, and concentrated under reduced pressure to give a residue, which was purified by HPLC (NP column, 300 × 8 mm, EtOAc-hexane (9:1), flow rate: 1.0 ml) to afford **1a** 20(*S*), 24(*R*)-epoxi-1β, 3α,12β, 25-tetrahidroxidammarane, 24-*epi*-polacandrin^14^ (7 mg, 70% yield, Rf 0.35 in EtOAc).

*Acetylation of* **1**. Compound **1** (25 mg) was dissolved in a mixture of pyridine (1 ml) and Ac_2_O (3 ml) with a catalytic amount of DMAP, and the mixture was heated under reflux in a glycerine bath for 3 h. The reaction mixture was then concentrated under reduced pressure and, after the usual workup procedure and purification by HPLC (NPcolumn, 300 × 8 mm, EtOAc-hexane (7:3), flow rate: 1.0 ml), gave the peracetylated compound **1b** 1β,3α, 12β25-tetracetoxi-20(*S*), 24(*R*)-epoxidammarane^14^.

### α-Synuclein expression and purification

The sequence of WT α-synuclein inserted into plasmid pT7-7 was donated gently by Dr. Claudio Fernández (Rosario, Argentina). This fragment was cloned into the pET-28a vector (Novagen) to produce a His-tagged protein expressed in *Escherichia coli* strain BL21 (DE3). LB medium containing kanamycin (30 µg/ml) was inoculated with a stationary overnight culture. The culture was grown at 37 °C to OD 600 of 0.4–0.6, and protein expression was induced by the addition of 1 mM IPTG for 4 h. The cells were pelleted and sonicated. Recombinant α-synuclein was purified via ProPac IMAC 10 (Thermo Fisher Scientific, Waltham, MA) using a gradient of 10–200 mM imidazole, 20 mM Na_2_HPO_4_, and 500 mM NaCl. The purity of the protein was verified on a Coomassie Brilliant Blue-stained SDS-polyacrylamide gel. The protein was concentrated and stored at −80 °C until use. The concentration was determined using the extinction coefficient at 275 nm (5600 M^−1^ cm^−1^) as described^15^.

### α-Synuclein aggregation and ThT measurements

Thirty micromolar of α-synuclein in 25 mM Na_2_HPO_4_ were incubated at 37 °C in constant shaking at 300 rpm for 48 h into Eppendorf tubes to form aggregates. To ThT fluorescence measurement concentration of ThT was adjusted to 25 µM, after 1 h of incubation at 37 °C, the plates were read in a Biotek H1 multi-mode reader in a 96-black plate flat bottom. The Ex/Em was set at 440/485 nm.

### Microscale thermophoresis

Microscale thermophoresis assays were performed on a Premium MonolithTM NT.115 microscale thermophoresis device (NanoTemper, Munich, Germany) after labelling the protein RED-NHS dye as specified by the manufacturer. Assays were performed in 50 mM phosphate buffer (pH 8.0) supplemented with 0.05% Tween-20 using 20 nM labelled protein and inhibitor concentrations varying between 750 µM and 0.02 µM. Samples were incubated 10 min at room temperature before loading the assay capillaries. Data were analysed with PALMIST^16^.

### Raman spectra acquisition and spectral processing

The samples, 3 µl, were placed on a slide and left to dry at room temperature. The acquisition was made through a Raman confocal microscope equipped with a laser with a wavelength of 532 nm and a CCD camera cooled to −60 °C. A single-mode optical fibre was used to connect the laser to a ×100 lens. The laser power was set to 10 mW. Each spectrum was obtained by averaging 10 accumulations with an acquisition time of 30 s for each accumulation, using WiTec's Control Four software. The scanned images, a matrix of 20 dots per line was established on a surface of 5 µm (width) and 5 µm (height), with an integration time of 5 s per spectrum. The spectral processing was performed using Matlab. The bands' intensities are normalised to the 1100 cm^–1^ bands' average intensity corresponding to the glass. Then, the spectral contribution of the glass was subtracted numerically. The Raman intensity was analysed by filtering peaks through the Lorentzian function. Raman acquisition was repeated in triplicate.

### AFM images in tapping mode

AFM imaging was performed in tapping (intermittent contact) mode using a Nanoscope III (Veeco, Santa Barbara, CA) and Si_3_N_4_ cantilevers (NPS series, Veeco, Santa Barbara, CA) exhibiting spring constants of 40–60 N/m at resonance frequencies of 6–10 kHz as described^17^. Briefly, α-synuclein 30 µM was diluted in 10 mM Tris–HCl, pH 7.4, 50 mM KCl, and then immobilised onto mica freshly cleaved to a final concentration of 5 µM. A drop of α-synuclein protein solution was adsorbed for 30 min. The excess of protein was removed by washing with 10 mM Tris–HCl, pH 7.4, and 50 mM KCl solution.

### N2a cell culture

N2a neuroblastoma cells were cultured at 37 °C in a humidified atmosphere at 5% CO_2_, using MMM Group^®^ Stove, Standard CO_2_ Cell 190, in DMEM D1152 plus F-12 medium supplemented with 10% bovine foetal serum^®^ Biowest, 100 µg/ml ampicillin and 100 µg/ml streptomycin, in T25 cell culture flasks. To the differentiation procedure, cells were cultured in Petri dishes 35/10 mm over coverslips, then was added SFB at 2.5% plus retinoic acid of 10 µM for five days replacing medium culture every 48 h for neurite grown monitored through an inverted microscope. Either aggregates alone or aggregates treated with **1** at 30 nM were added at day 4 for 24 h in the culture medium. After that, cells were fixed for immunofluorescence. The cells were then washed twice with 1× PBS buffer (137 mM NaCl, 10 mM Na_2_HPO_4_, 2.7 mM KCl, 1.76 mM KH_2_PO_4_), fixed for 30 min with 4% paraformaldehyde at room temperature, washed three times with PBS, and permeabilised for 10 min with Triton X100 0.5% (Sigma-Aldrich, St. Louis, MO) diluted in PBS. Finally, coverslips were washed three times with PBS. After that, cells were incubated with a 1% BSA (Sigma-Aldrich, St. Louis, MO) for 30 min at room temperature. Subsequently, samples were incubated at 37 °C for one hour with the supernatant of α tubulin monoclonal TU-01 kindly donated by Dráber and coworkers^18^^,^^19^, then diluted in blocking solution 0.1% BSA and washed three times in PBS. After that, cells were incubated with Alexa Fluor 488 Goat anti-Mouse IgG (Thermo Fisher Scientific, Waltham, MA) for one hour at room temperature and washed three times in PBS. Subsequently, the cells were mounted 4′,6-diamidino-2-phenylindole (Vectashield, Vector, Burlingame, CA). Samples were analysed in a confocal microscope, Zeiss LSM Meta (Oberkochen, Germany).

### Molecular docking

The structure of full-length α-synuclein fibrils has been resolved by solid-state NMR (protein data bank code 2N0A)^20^. This model contains 10 units of the peptide (140 amino acids), which are arranged in parallel by presenting features that commonly stabilise amyloid folds (such as intermolecular salt bridge, a glutamine ladder, steric zippers involving hydrophobic residues, and in-register parallel-β-sheet hydrogen-bonding with a Greek key motif). In a recent report, Hsieh et al. used this structure as a target protein for molecular blind docking of small molecules^21^. They found 13 putative binding sites, but they excluded a number of these sites based on their low percent probability of interaction; finally, they identified site 2 (with residues Y39, S42, and T44), site 9 (with residues G86, F94, and K96), and the site 3/13 (with residues L43, L45, V48, and H50)^21^. Docking calculations were executed inside these sites by using Glide XP method^22^. The structures of **1**, **1a**, and **1b** were sketched using Maestro’s molecular editor (Maestro 10.2.011, Schrödinger LLC, New York, NY). Grid boxes of 25 Å×25 Å×25 Å were defined in the centre of these sites. Docking parameters were used as in previous works^23^^,^^24^. The docking protocol begins with the systematic ligand conformational search followed by its placement in the receptor sites. Minimisation was performed by using the OPLS-AA^25^ force field (distance-dependent dielectric = 2.0). Further, a Monte Carlo algorithm samples the nearby torsional minima of the lowest energy poses. According to the energies ranked by GlideScore, the more favourable conformations were selected as the best poses.

The stability of the identified docked poses was done using molecular dynamics (MD) simulation with the model of α-synuclein fibrils forming complexes with compound **1** (3 units) in sites 2, 9, and 3/13, respectively, since compound **1** was the most potent inhibitor. Desmond v3.5 software was used with the OPLS-AA force field^25^^,^^26^. The complex was solvated to construct a 112 Å×140 Å×100 Å water box. Na^+^ and Cl^–^ ions were added to replicate physiological conditions and guarantee that the whole system was neutral. Simulation parameters were established as described here: an NPT ensemble was used, the constant temperature at 300 K was imposed by using the Nosé–Hoover chain thermostat, and constant pressure at 1 atm was imposed by using the Martyna–Tobias–Klein barostat. RESPA integrator was applied with a 2-fs time step for bonded and nonbonded-near forces and 6 fs for long-range forces. Particle-mesh Ewald with a 9 Å cut-off radius was employed for long-range electrostatics. The protein backbone was restrained with a constant force of 5 kcal mol^−1^ A^−2^. In the beginning, a 20-ns MD simulation was performed to equilibrate the system, and then the production MD was performed for 150 ns.

### Data analysis

The Raman spectra analysis was done by using Matlab software (Natick, MA), and the statistical analysis was done by using GraphPad 7 (La Jolla, CA).

## Results

### Effect of 1 over α-synuclein aggregation

**Figure 1.**
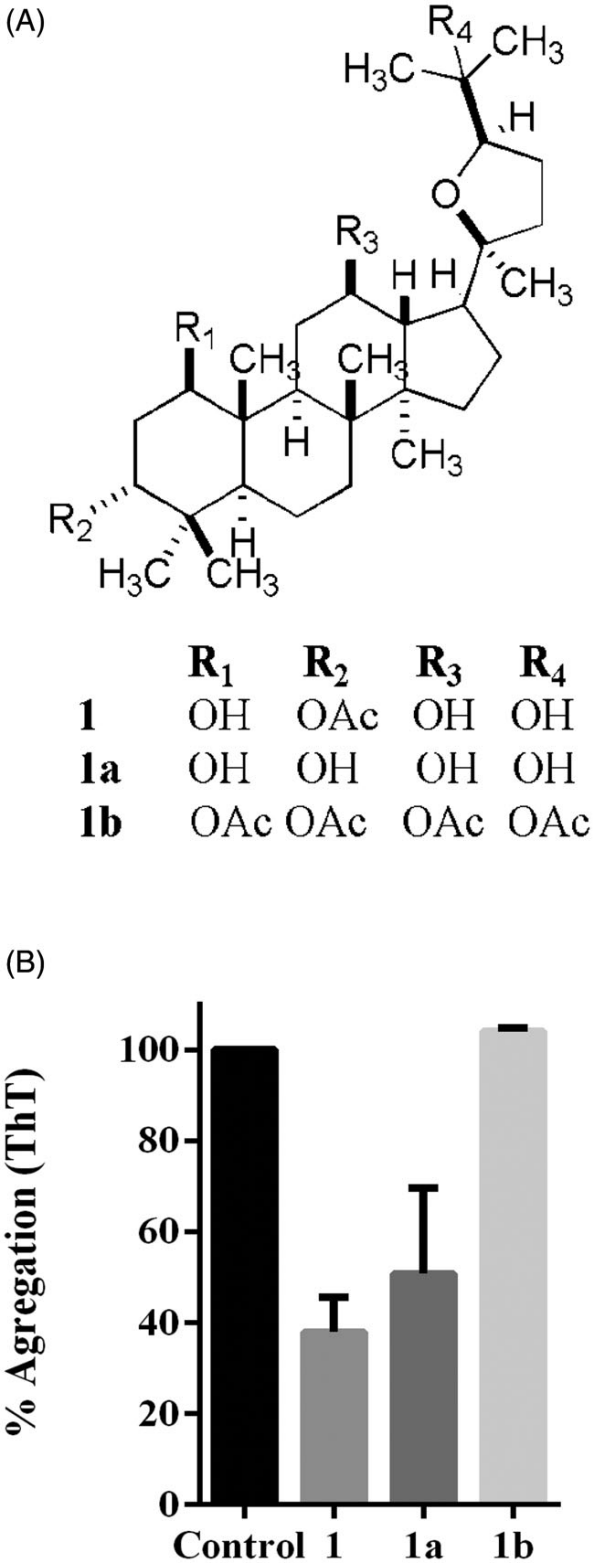
(A) Compound **1** is 3α-acetoxi-20 (*S*), 24 (*R*)-epoxi-1 β, 12 β, 25-trihidroxidammarane, named 3 α-acetil-24-*epi*-polacandrin **1a** is the hydrolysis product of **1**, and compound **1b** is its peracetylated derivative. (B) Thioflavine T experiment at 48 h. Significance was determined with ANOVA with Dunnett's test at *p<* 0.05.

**Figure 2.**
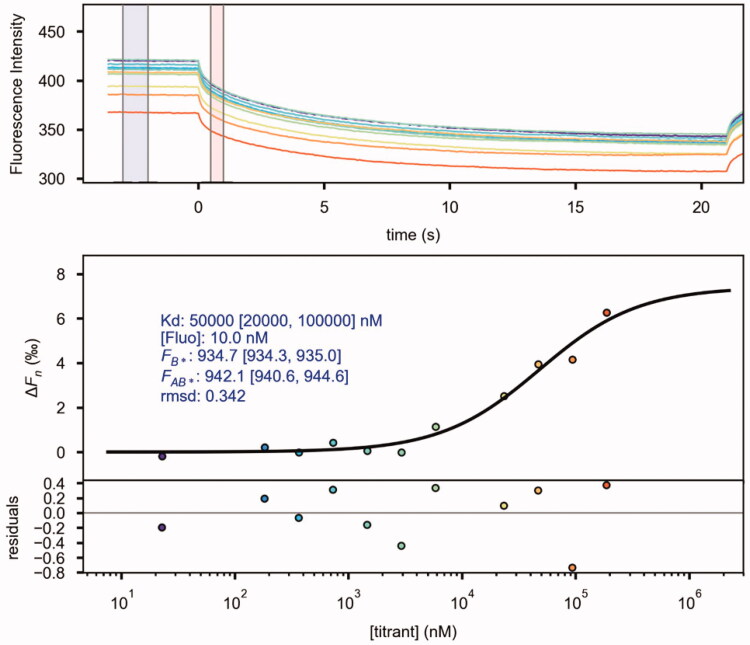
Dissociation constant (Kd) measured in the presence of **1**. (A) Graphical representation of time versus fluorescence. (B) Dose–response graph of the corresponding thermophoresis measurements shown in (A).

The isolation of **1**, and further derivatives **1a**, **1b** (Figure 1(A)) were tested as α-synuclein inhibitors through fluorescent probe thioflavine T, which has a high affinity for fibrils amyloids^27^. Thus, we decided to induce aggregation for 48 h of α-synuclein. Then, we assayed the three compounds against fibril formation. Thus, compounds **1** and **1a** at 100 µM shown inhibition activity into the assembly of β-sheet since there is a diminishing in fluorescence intensity. Interestingly, the peracetylated compound **1b** did not exert inhibition against the assembly of β-sheet, suggesting that hydroxyls and acetyl group in compound **1** play a role in the inhibition of fibril formation (Figure 1(B)). Thermophoresis (TMS) is known as the Soret effect, and it is an analytical method introduced as an alternative to studying interactions between protein and small compounds^28^. The Soret effect corresponds to the mobility of the particles under the influence of a gradient of temperature. Thus, we decided to determine the interaction between α-synuclein and **1**; our results of the dissociation constant (Kd) was 50 µM, considering that this technique was designed to study non-covalent interactions such as hydrogen bonds that might be involved in the interaction between α-synuclein and **1** (Figure 2(A,B)).

The aggregation process reveals morphological and amide region pattern changes after treatment with **1**.

**Figure 3.**
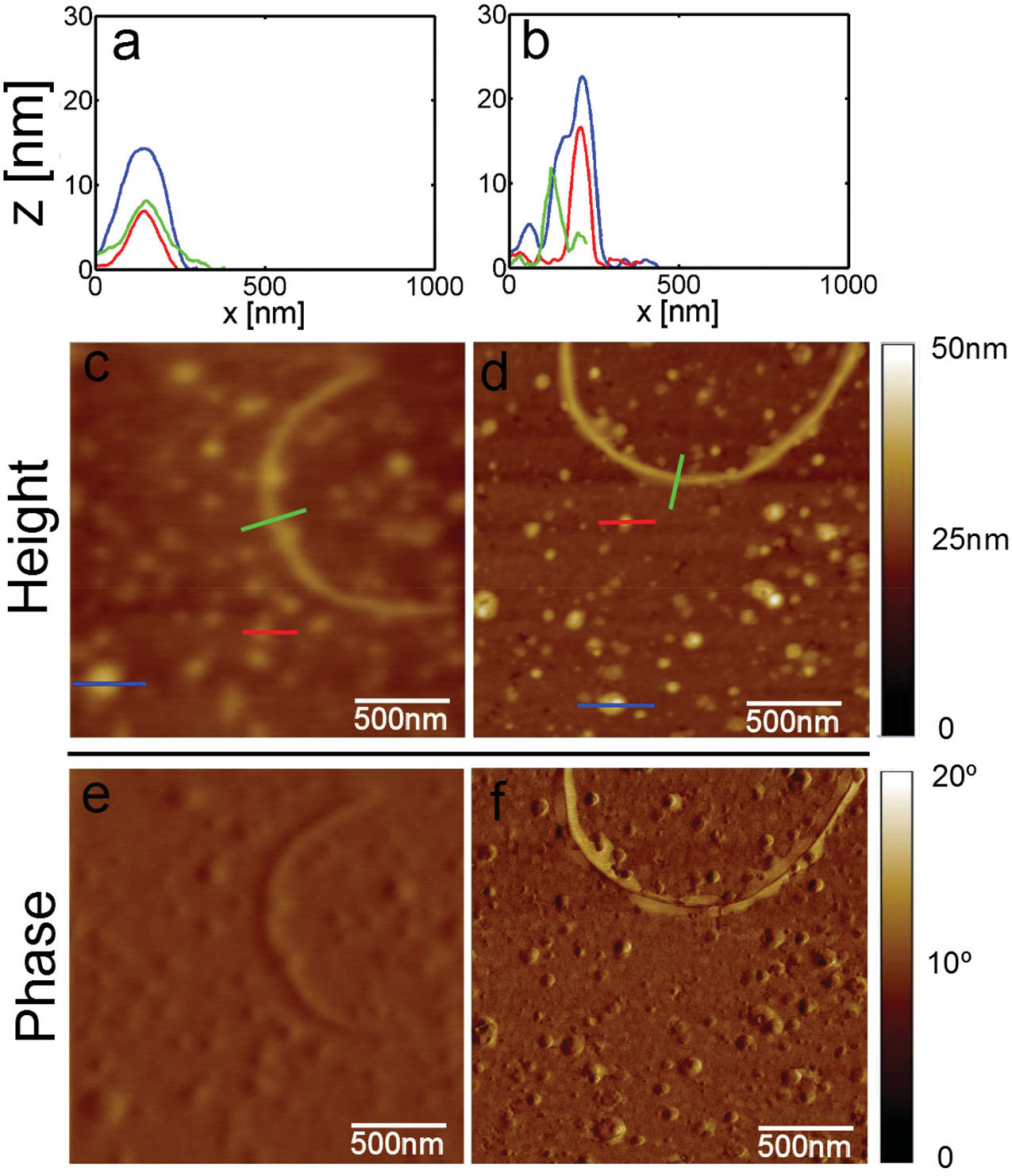
Atomic force microscopy images of α-synuclein aggregates represented as control of aggregation, height, and phase are depicted.

**Figure 4.**
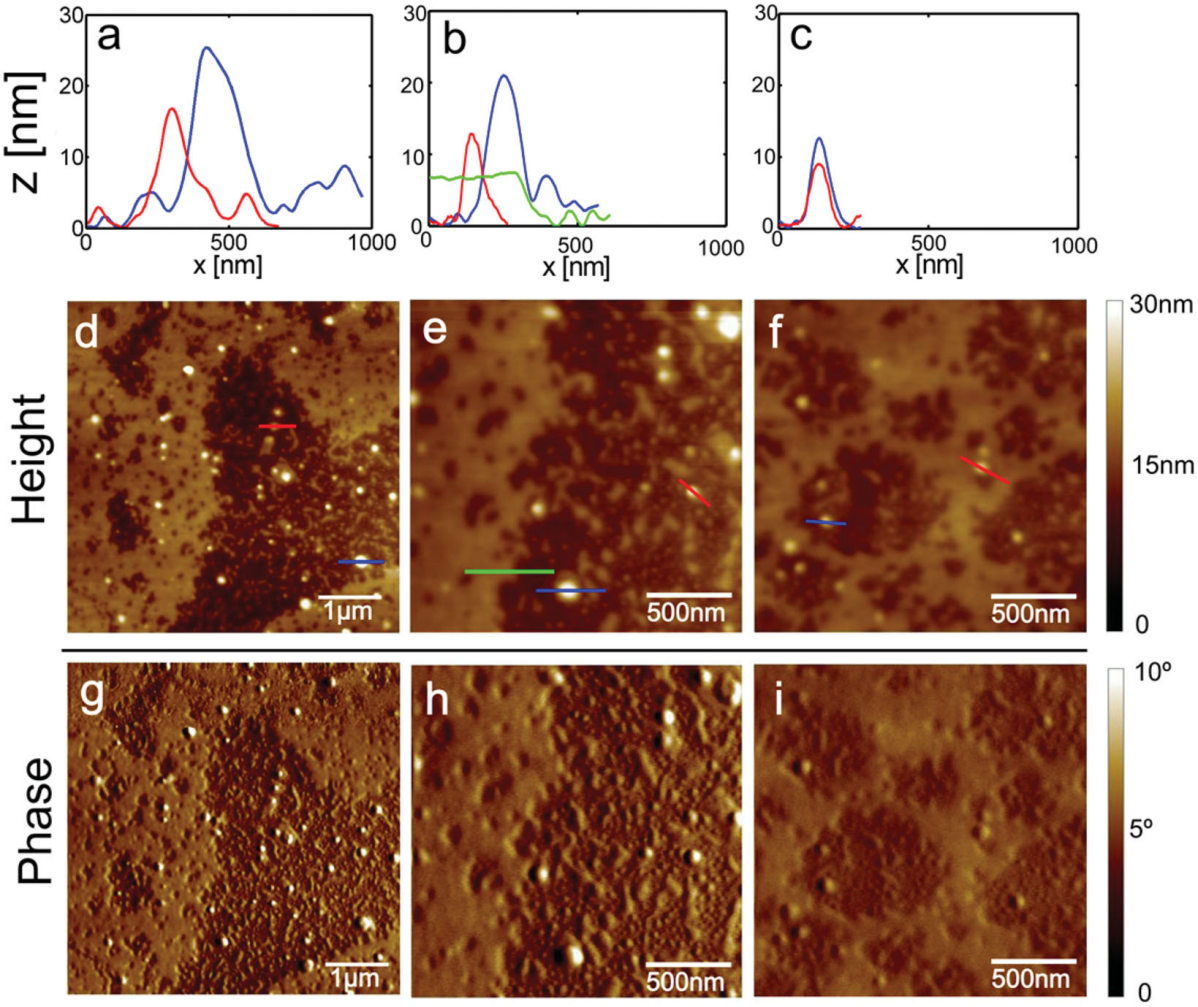
Atomic force microscopy images of α-synuclein aggregates in the presence of **1**, height, and phase are depicted.

Then, we performed atomic force microscopy (AFM) experiments to corroborate the effect of **1** on α-synuclein aggregation. The tapping mode on mica provides the phase information, making it possible to distinguish morphological differences between the samples. Besides, AFM images in the tapping mode allow for the determination of topographic details, verifying its thickness using a cross-section obtained from the topographic image showing the distribution of elements. In control experiments, elements such as oligomers and fibrils are presents, whose height is about 15 nm, while in treated aggregates, the height is about 5 nm. Moreover, the diameter of treated aggregates is ranging from 180 to 400 nm. However, the control experiment aggregates have a diameter distributed under 200 nm (Figures 3 and 4 and Supplementary Figure S1), suggesting different pathways in these aggregates' conformation.

**Figure 5.**
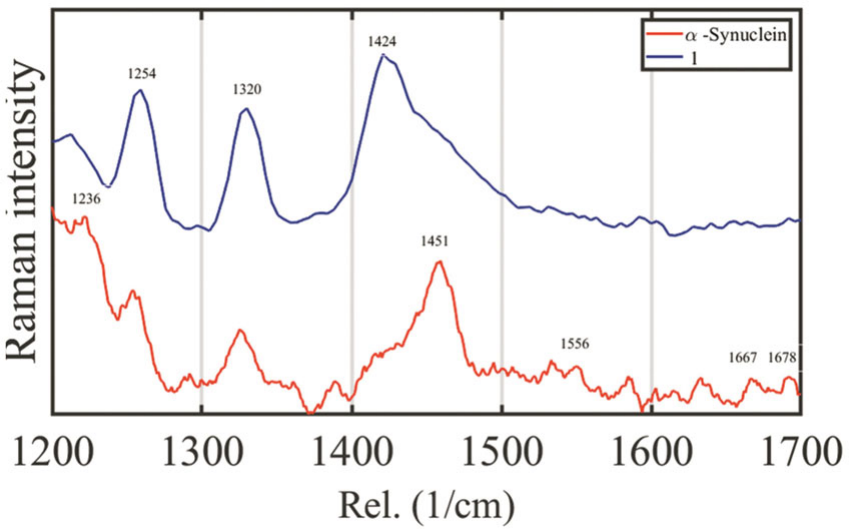
Raman spectroscopy of α-synuclein after 48 h of incubation in the absence or presence of **1**. Raman spectra were recorded, including regions amide I, II, and III, in the control of aggregation (red spectra) and only amide III in α-synuclein (blue spectra).

Raman spectroscopy experiments reveal that upon α-synuclein aggregation, we observed bands belonging to the amide I region related to C═O stretch at 1667 cm^−1^, associated with β sheet content. It also arises from the same region, a band at 1674 cm^−1^ belonging to polyproline II^29^. In the amide region II related to C–N stretch and N–H bend, there is a band at 1556 cm^−1^ associated with β-sheet content. In the amide region III, whose vibrational modes are associated with C–N stretch and N–H bending, a band at 1236 cm^−1^ arises linked with a β-sheet secondary structure. There are two bands at 1320 and 1451 cm^−1^ related to CH_2_ deformation and CH_2_ and CH_3_ deformation, respectively, as previously described^29^. Once we analysed the Raman spectra of α-synuclein in the presence of **1**, we found bands most associated with the amide III region, which includes bands at 1320 and 1424 cm^−1^ representing CH_2_ deformation and CH_2_ and CH_3_ deformation, respectively. Also, we found a massive band at 1254 cm^−1^ that mostly represents random or unfolded protein. Thus, once we found a typical distribution representing α-synuclein aggregates, including bands associated with β-sheet, and the case of α-synuclein treated with **1** remains unfolded (Figure 5).

N2a cells preserve their neurite outgrow in the presence of treated aggregates by **1**.

**Figure 6.**
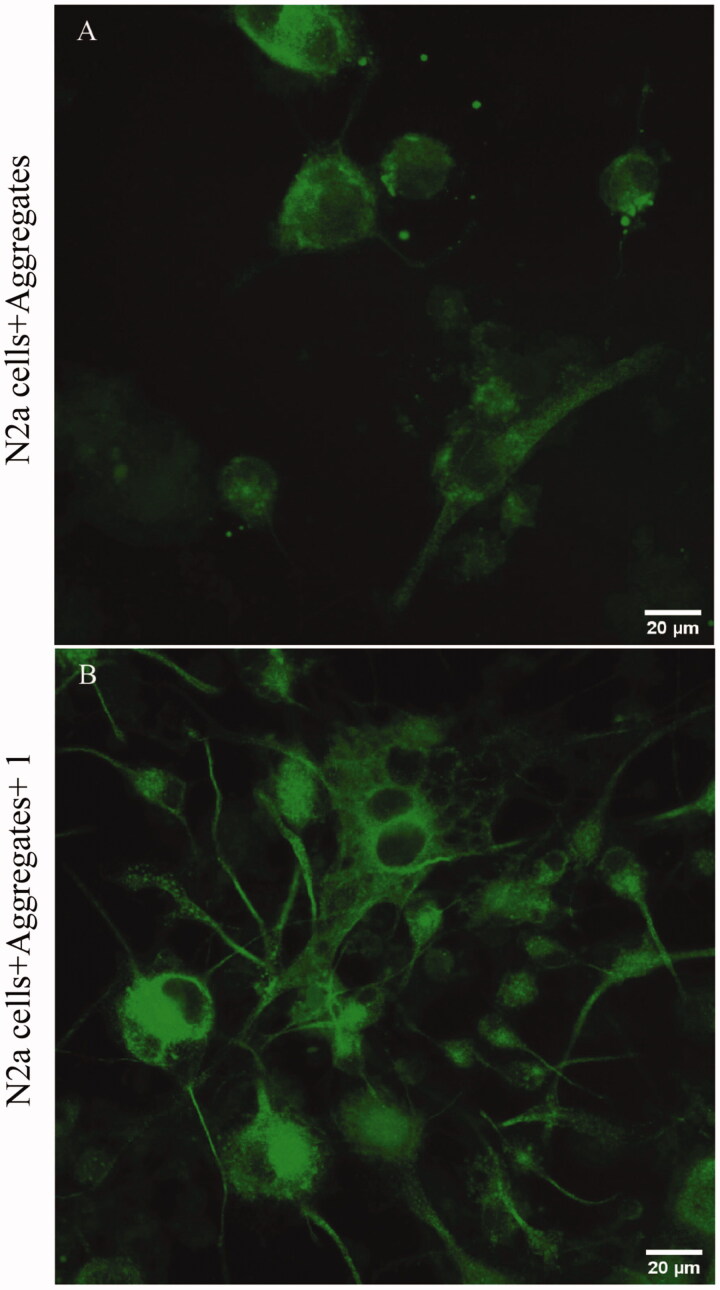
(A) α-synuclein aggregates (30 nM) incubated for 24 h in previously neurite-like structure induced by retinoic acid in N2a cells. (B) α-synuclein aggregates +**1** (30 nM) incubated for 24 h in previously neurite-like structure induced by retinoic acid in N2a cells. Bars 20 µm.

Several pieces of evidence show that aggregates of α-synuclein could severely compromise the dopaminergic terminal and axons, leading to cell death^30^. Therefore, we decided to investigate whether externally added aggregates to cell culture could provoke damage to N2a cells. Thus, we induced neurite extension through retinoic acid to resemble neurite-like outgrow (Supplementary Figure S2). Then, we incubated extracellular aggregates treated or not of compound **1** at 30 nM. Further, we observed that in N2a cells in the presence of α-synuclein aggregates, most of the cells retract their neurite extension and provoke cell swelling (Figure 6(A)).

Further, once we challenged N2a cells in the presence of the aggregated treated with **1**, the cells mostly preserve their neurite like-structure, as shown in Figure 6(B).

### Elucidation of binding sites by 1 through molecular docking

**Figure 7.**
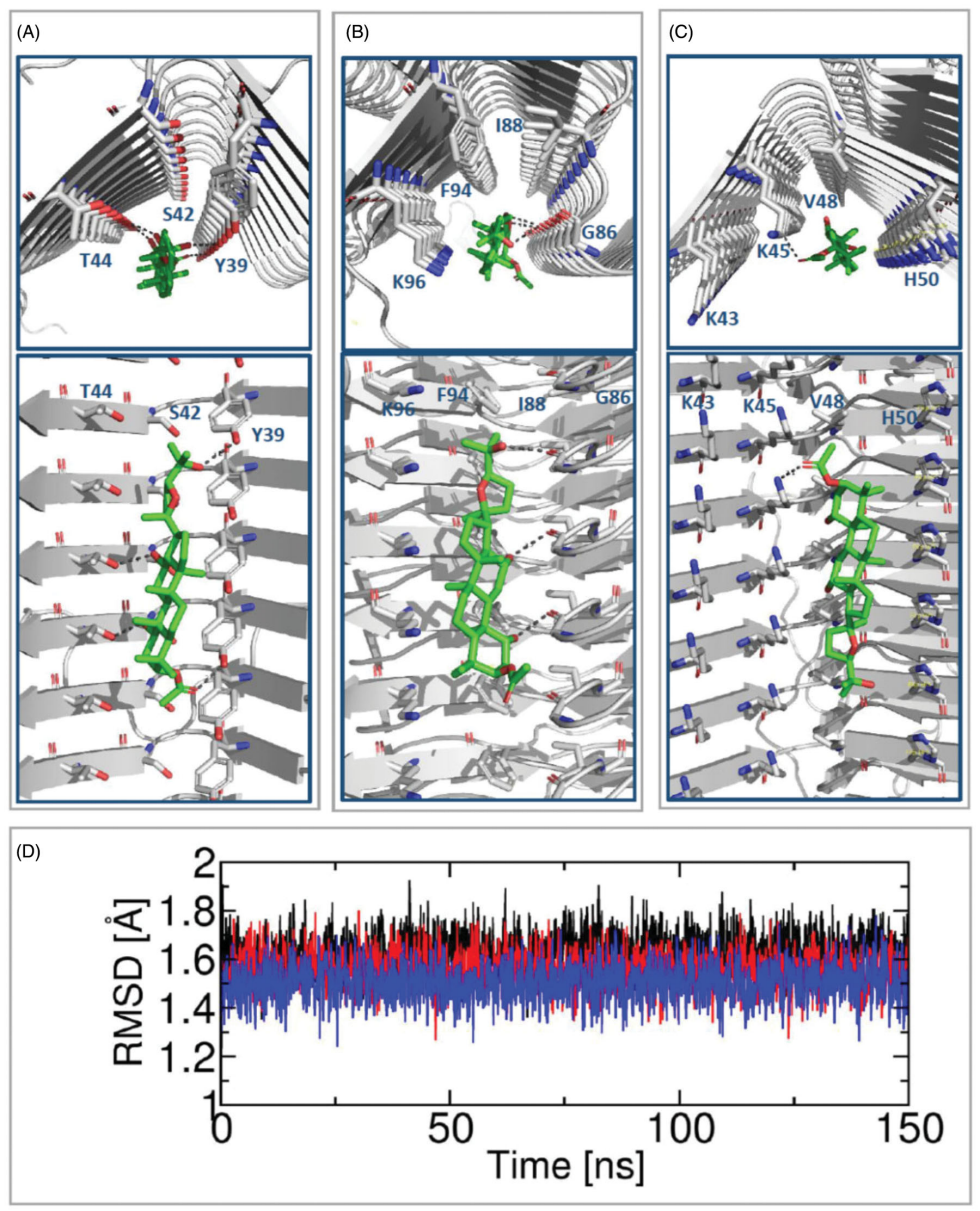
Molecular modelling studies of **1** forming complex with sites of α-synuclein. (A) Docking in site 2; (B) docking in site 9; (C) docking in site 3/13 (docked poses are viewed from the fibril axis at the top and are viewed from the fibril side view at the bottom, α-synuclein is in the grey cartoon). (D) RMSD of **1** in the sites 2 (black), 9 (blue), and 3/13 (red) from a 150-ns MD simulation.

Finally, to elucidate the potential binding sites, the docking results for **1** inside sites 2, 9, and 3/13 of α-synuclein fibrils are shown in Figure 7(A–C). The obtained models suggest that **1** is capable of binding to these sites. However, the scoring energies are more favourable for the interaction with site 2 (scoring energies for the sites 2, 9, and 3/13 had values of ‒4.617, ‒2.545, and ‒2.592 kcal/mol, respectively). The most energetically favourable docking pose of **1** extends along with site 2, where hydroxyl and carbonyl groups of the ligand form hydrogen bonds with the side chains of several Y39 and T44 residues (Figure 7). Docking experiments revealed that small compounds could display preferences for different sites^21^. The docking poses' stability was tested by performing a 150-ns MD simulation of the system that contains the three sites occupied by compound **1**. This simulation revealed that compound **1** remained stable in the three sites since root-mean-square deviations (RMSDs) had almost constant values during the MD trajectory (Figure 7(D)). Our results indicate that **1** fit into the three explored sites since it has a good shape and can form stable chemical interactions.

Atomistically, the destabilisation of toxic α-synuclein oligomers by interacting with small molecules should proceed by complex mechanisms^31^. In these mechanisms, it is reasonable that the first step is to recognise the small molecule in sites of α-synuclein fibrils. Our docking results for complexes between dammarane triterpenes and α-synuclein fibrils represent the possible recognition sites that initiate this process. The elucidation of the complete mechanism, produced on large timescales, should consider that disruption of the fibrils' integrity induces significant conformational changes in the protein framework.

The docking poses for derivatives **1a** and **1b** inside the sites 2, 9, and 3/13 of α-synuclein fibrils were also obtained to explain the more significant inhibitory activity of compound **1**. The energies of binding and the description of the α-synuclein residues that form hydrogen bond interactions with these compounds are depicted in Supplementary Table S1 and Figure S3. Docking results revealed that compound **1** had the most favourable energy value in site 2 (with scoring energy of ‒4.617 kcal/mol as mentioned above). In comparison, compounds **1a** and **1b** had scoring energies of ‒4.286 kcal/mol (inside site 9) and ‒3.702 kcal/mol (inside site 2). The calculated energy trend is in agreement with the experimental activity trend. The analysis of the complexes' structural features, specifically the intermolecular hydrogen bonds, allows explaining the differential activity of the three dammarane triterpenes **1**, **1a**, and **1b**. The substituents in compound **1** (R_1_=OH, R_2_=OAc, R_3_=OH, and R_4_=OH) can establish hydrogen bonds with two Y39 and two T44 residues inside site 2 (Figure 7). When OH replaces OAc in R2 in compound **1a**, and when OAc replaces OH in R4 in compound **1b**, one of the hydrogen bond interactions with T44 is lost. This analysis indicates that compound **1** has optimal substituents for establishing hydrogen bond interactions with α-synuclein fibrils.

## Discussion

Among the neurodegenerative disorders, PD is the fastest-growing neurodegenerative disorder after Alzheimer's disease affecting older people worldwide. The global burden is around 6.2 million people affected, whose number will be increasing around 13 million by 2040^32^. The standard treatment is the use of l-DOPA; however, there are several side effects. PD's pathological hallmark is α-synuclein aggregates, whose NAC region involves a hydrophobic region responsible for its fibrillisation^33^. In this regard, several compounds interfere in different ways, either stabilising the monomer or preventing the fibrillisation^31^. Thus, avoiding the formation of these aggregates appears as an alternative for drug development.

Interestingly, a polyphenol (–)-epigallocatechin gallate redirects the amyloid pathway preventing the formation of structures rich in β-sheet conformation through hydrogen bonds^34^. Moreover, these non-toxic elements do not provoke cytotoxicity in PC12 cells^13^. Moreover, baicalein, a natural flavonoid, can prevent fibrillisation and disassembly of existing fibrils through the interaction of o-quinone with lysine forming a Schiff base^34^. Interestingly, baicalein can protect dopaminergic neurons in mice^35^^,^^36^.

Besides, some compounds containing a nitro-catechol moiety arise as alternatives for PD treatment by slowing dopamine degradation^9^. Moreover, compounds such as entacapone and tolcapone inhibit fibrillisation of α-synuclein, preventing aggregates' extracellular toxic effect on PC12 cells^37^.

Here, we showed in the ThT experiment that compound **1** could inhibit β-sheet assembly through non-covalent interactions such as hydrogen bonds, confirmed by TMS experiments. Since this technique arises to quantify and determine the dissociation constant (Kd) through a temperature gradient, we determined this value in 50 µM.

The structure we observed in AFM experiments correspond to fibrils and rounded morphological elements resembling oligomers. In the presence of **1**, we found that the diameter ranges from 180 to 400 nm, suggesting that an accumulation of elements could occur instead of forming fibrils. The Raman spectra of aggregation control reveal that most of the amide signals are related to the β-sheet formation and CH_2_-CH_3_ deformation. In the case of treated aggregates in the presence of **1**, we found most spectra related to unfolded α-synuclein and CH_2_-CH_3_ deformation, suggesting that those elements could correspond to an off-aggregation pathway. It is crucial to considerer that neurite outgrow is essential for cells. Thus, to test the hypothesis that the aggregates treated with compound **1** are non-toxic to cells, we decided to resemble this process by inducing neurite-like structures in the presence of retinoic acid. Then, we externally added to the cell culture non treated and treated aggregates by **1**. In cells challenged with non-treated aggregates, most of them retract neurite outgrow, and the presence of non-treated aggregates provokes cell swelling. In the cells challenged with treated aggregates in the presence of **1**, the neurites remain to suggest a non-harmful effect over cell culture. Finally, docking simulation reveals that **1** prefers sites 2, 9, and 3/13 in the NMR solid-state α-synuclein structure.

## Supplementary Material

Supplemental Material
