# Supplementary material for: Dammarane triterpenes targeting α-synuclein: biological activity and evaluation of binding sites by molecular docking
Source: J Enzyme Inhib Med Chem. 2020 Dec 14;36(1):154–62. doi: 10.1080/14756366.2020.1851216 (PMC7738290; doi:10.1080/14756366.2020.1851216)
Supplement: Supplemental Material [file IENZ_A_1851216_SM6699.pdf]

Supplementary material

Dammaranes triterpenes targeting α-synuclein: biological activity and evaluation of binding sites by molecular docking

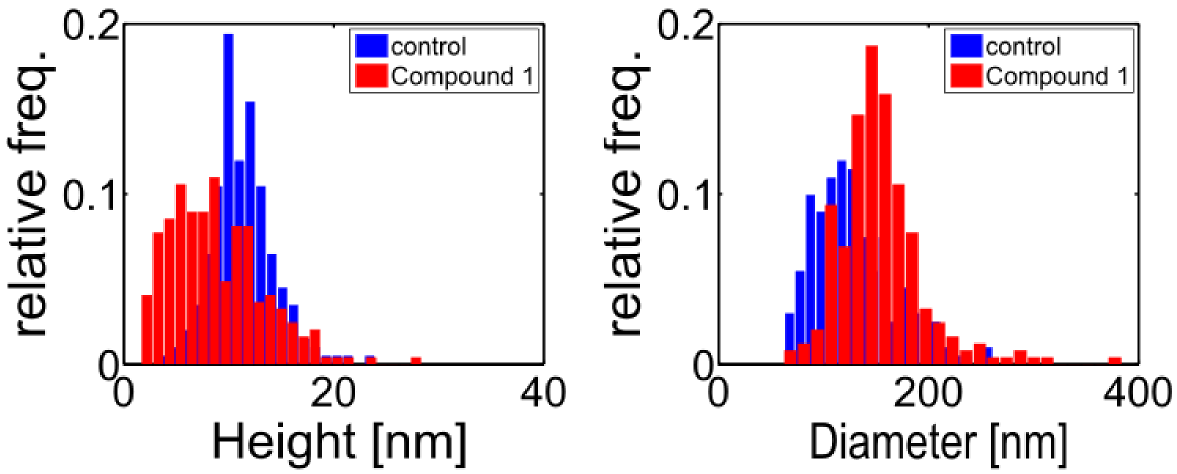

Figure S1. Histogram relative frequencies of aggregates in the presence or not of **1**.

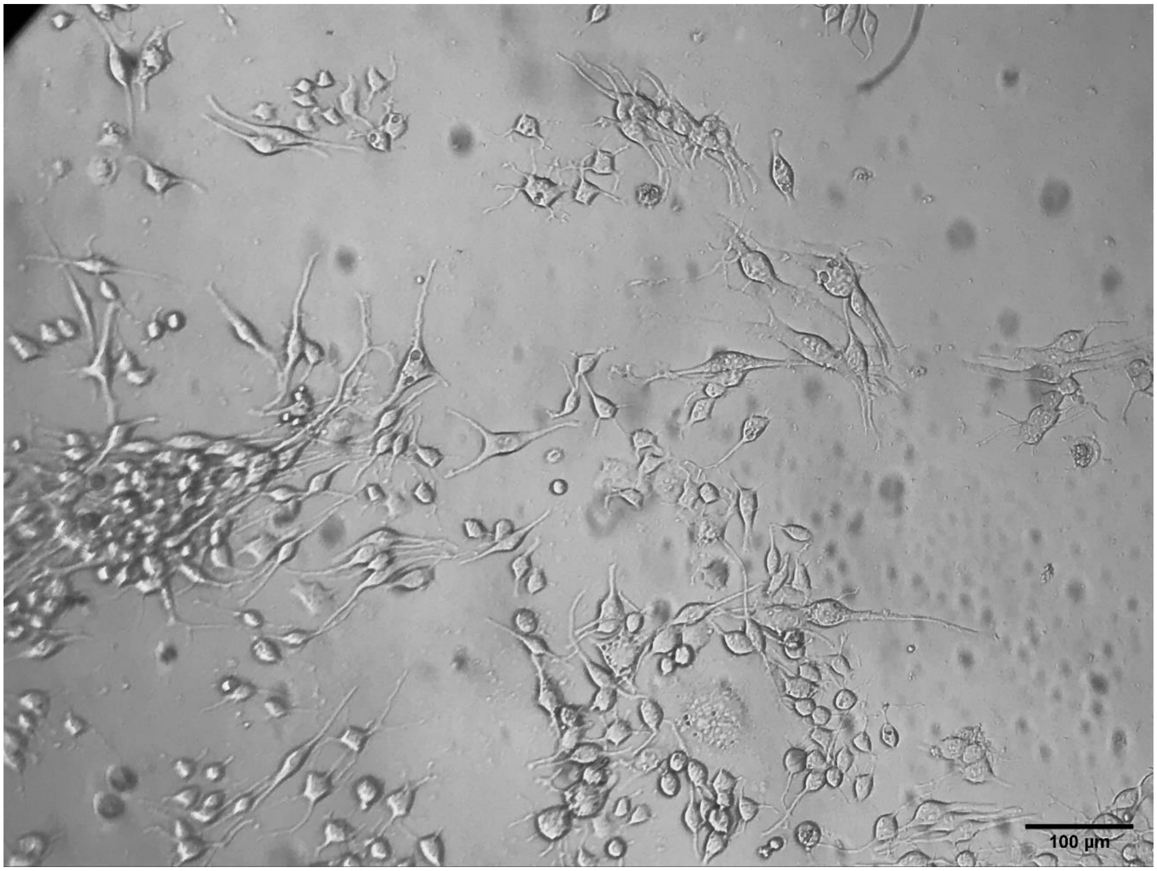

Figure S2. Bright-field image of N2a cells (control) induced with retinoic acid for four days to induce a neurite-like structure before incubating for 24 h with aggregates and aggregates treated with compound **1.** Bar 100 µm.

Table S1. Energy values of the docked poses of compounds **1**, **1a**, and **1b** and α-synuclein residues are hydrogen-bonded to them. [a]

Compound	α-synuclein site	Glide XP Scoring Energy (kcal/mol)	Residues of α-synuclein that are hydrogen-bonded to the docked pose.
**1**	Site 2	**-4.617**	Two residues Y39 and two residues T44
	Site 9	-2.545	Two residues G86
	Site 3/13	-2.592	One residue K45
**1a**	Site 2	-3.495	Two residues Y39 and one residue T44
	Site 9	**-4.286**	Three residues G86 and one residue K96
	Site 3/13	-3.002	One residue H50
**1b**	Site 2	**-3.702**	Two residues Y39 and one residue T44
	Site 9	-2.900	Four residues K96
	Site 3/13	-2.830	Two residues K45

The most favorable energy value for each compound is in bold letters.

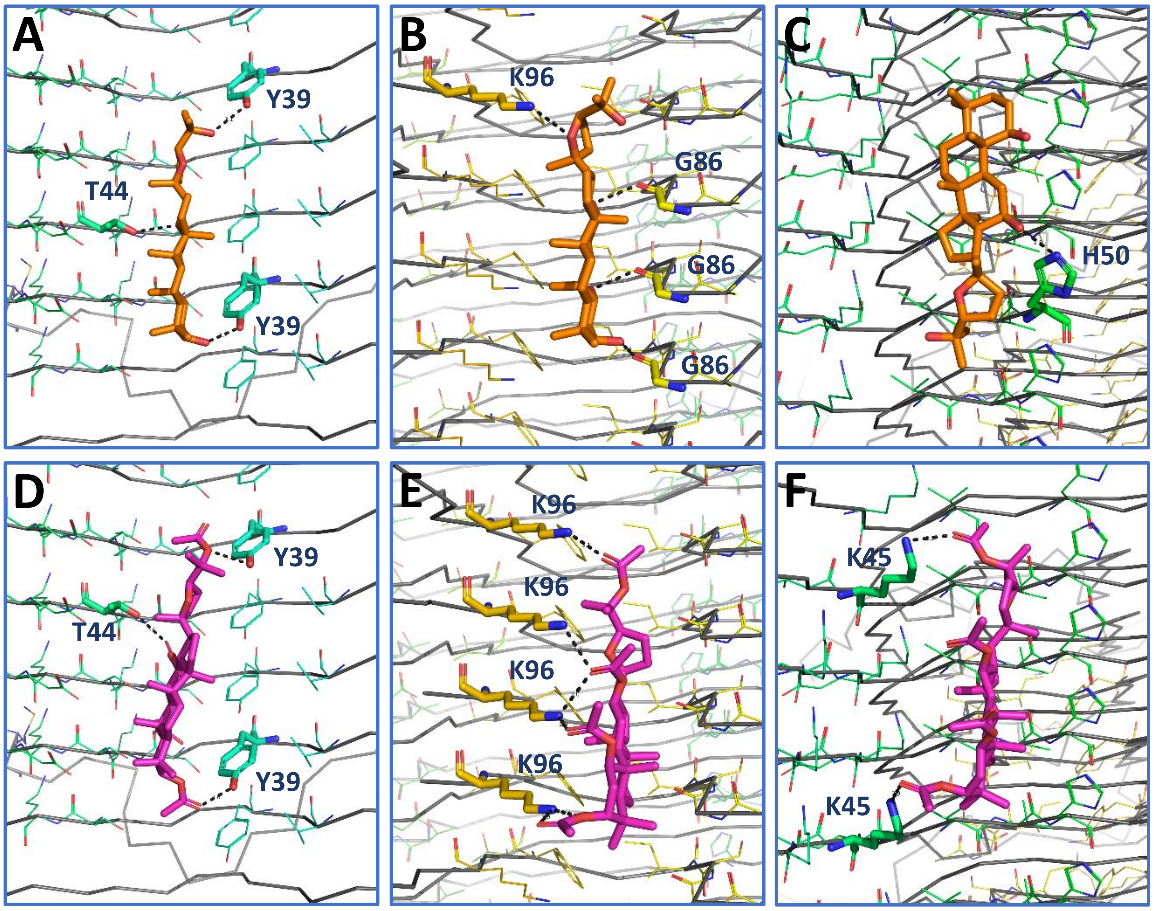

Figure S3. Molecular modeling studies of **1a** and **1b** forming complexes with sites of α-synuclein. Docking poses of **1a** in sites 2 (A), 9 (B), and 3/13 (C) are in orange sticks. Docking poses of **1b** in sites 2 (D), 9 (E), and 3/13 (F) is in purple sticks. Residues that form a hydrogen bond with the compounds **1a** and **1b** are depicted.
